# Transcriptome profiling of guinea pig skin exposed to a high‐power terahertz source

**DOI:** 10.1002/em.22470

**Published:** 2022-01-03

**Authors:** Chengxia Zhou, Lidan Xiong, Xun Zhou, Li Li, Qiang Yan

**Affiliations:** ^1^ Department of Dermatology West China Hospital, Sichuan University Chengdu China; ^2^ Cosmetics Safety and Efficacy Evaluation Center West China Hospital, Sichuan University Chengdu China; ^3^ NMPA Key Laboratory for Human Evaluation and Big Data of Cosmetics Chengdu China; ^4^ Sichuan Engineering Technology Research Center of Cosmetic Chengdu China; ^5^ Research Center of Laser Fusion, CAEP Mianyang China

**Keywords:** guinea pig, high‐power terahertz, RNA‐seq, skin, transcriptome profiling

## Abstract

Terahertz (THz) radiation has a wide range of applications including use in medicine. However, effects of high‐power THz radiation have not been clearly elucidated. We used a 2.52 THz self‐made optically pumped gas THz laser, the low‐ and high‐energy group, to irradiate the backs of Hartley guinea pigs. RNA‐sequencing was done to explore global transcriptional responses in the irradiated skin. Gene Ontology analysis revealed that differentially expressed genes (DEGs) between the unexposed and low‐energy exposed groups were associated with skin development, skin barrier establishment, and multicellular organismal water homeostasis or water loss regulation via the skin. On the other hand, comparison between the unexposed and high‐energy exposed groups showed that the DEGs mediated monocarboxylic acid metabolism, blood vessel morphogenesis, establishment of skin barrier, blood vessel development, or angiogenesis. Our analyses demonstrate the potential effects of high‐power THz source on the skin and sets the basis for further studies on the safety and application of the high‐power THz in dermatology.

## 1 INTRODUCTION

The terahertz (THz) is an electromagnetic spectrum and entails frequencies from 0.1 × 10^12^ to 10.0 × 10^12^ Hz, with wavelengths in the range of 3 mm to 30 μm. The THz lies between microwave and far infrared regions. Until recently, there was no easy and direct process for generating THz sources whose strength are comparable to the powerful high‐energy lasers (Fedorov and Tzortzakis, 2020). The THz has a wide range of potential applications in areas related to imaging such as diagnostics, industrial quality control, security, food inspection, or artwork examination (Afsah‐Hejri et al., 2019; Cheng et al., 2021; Ohrstrom et al., 2015).

The THz has been used in the diagnosis of skin melanoma and nonmelanoma cancer, dysplasia, scars, diabetes, and could be utilized to evaluate the efficacy of different transdermal drug delivery systems (Hernandez‐Cardoso et al., 2017; Li et al., 2020; Nikitkina et al., 2021; Wang et al., 2020). Spectral responses of skin models with sweat ducts exhibited comparable behaviors as models without sweat ducts (You et al., 2018). Besides, portable THz spectrometer tools have been designed for noninvasive assessment of skin (Echchgadda et al., 2013).

Previous studies have demonstrated that THz from natural sources or artificial elements of low energy is safe for humans because of its low heat production and limited penetration. For instance, human skin cell expose to THz radiation from a 2.52 THz source did not induce genomic or DNA damage in the skin cells in vitro. Besides, whereas THz radiation on adult fibroblasts showed no alterations in expressions of proteins associated with DNA damage and repair, there was an increase in the frequency of centromere‐positive micronuclei as well as chromosomal nondisjunction events, (Franchini et al., 2018). With the advances in technology, there is invention of high‐energy THz. Therefore, there is need to analyze genotoxic properties and THz‐type phototoxicity of ultrahigh frequency electromagnetic fields of the THz range for human protection and safety.

In addition, given the widespread use of the THz, there could be an increase in the incidental and purposeful exposure of humans to high frequency ranges. Actin filament manipulation in living cells can be induced by 4 THz with a 1 THz bandwidth radiation without membrane injury or cell death (Yamazaki et al., 2020). Besides, previous studies showed that THz radiation on the skin could cause a rise in temperature by 3°C during the exposure, thus radiation (2.52 THz) generated thermal outcomes in mammalian cells (Wilmink et al., 2011). On the other hand, THz sources produce cellular responses against wound‐like stimulation. For example, there was stimulation of wound healing by TGF‐β‐induced synthesis of collagen after the radiation (Kim et al., 2013). This data implies a potentially beneficial application of the THz sources in skin regeneration.

Here, we developed a high‐power THz source, providing power density of 1.4 W/cm^2^ for the test to investigate potential skin changes in Hartly guinea pigs.

## 2 MATERIALS AND METHODS

### 2.1 High‐power THz source

We developed a THz source from an optically pumped gas THz laser Yan et al., 2017). The source was pumped by a CW single‐mode tunable CO_2_ laser and tunable across a wide THz spectrum and provided a maximum output power of over 200 mW. CH_3_OH was used as a THz laser medium and is responsible for more than 300 THz laser lines. The CH_3_OH gas was excited by the 9P (36) line (9.69 μm) of the CO_2_ pump laser, with transition from ground vibrationless state to a higher vibrational state. We obtained a 2.52 THz laser line with an output power of over 200 mW and a CH_3_OH pressure of 30 Pa. All the experiments in this study were conducted at 2.52 THz.

Besides, a 90° off‐axis parabolic gold mirror was used to focus the 2.52 THz beam. Its reflected focal length is 101.6 mm and a diameter of 50.8 mm, five times the input THz beam diameter. The focused beam propagates through a hard‐edged circular aperture with a diameter of 3 mm and approximately 70% of the THz energy passes through the small aperture. On the other hand, the THz laser beam radius was limited to 1.5 mm right after the aperture plane, where the guinea pig was exposed and the THz wave power was evaluated using a Scientech Vector Series H410 power meter.

### 2.2 Animals and tissue collection

Female guinea pigs and males are similar in skin structure, the females were less aggressive and scratched during feeding stage than males. Female Hartley guinea pigs with an average age of 10 weeks and a mean weight of 400 g from the Jiangsu ALF Biotechnology Co. were prepared. The guinea pigs were raised at temperatures between 20°C and 25°C and a humidity of 40%–60%, supplied with unlimited feed and clean water, which were changed once a day. The experiments were approved by the institutional ethical committee and performed in compliance with the institutional guidelines of West China Hospital.

Nine healthy guinea pigs were used for RNA sequencing, which were randomly assigned into three groups: low‐energy, high‐energy, and control groups. The back hairs of guinea pigs were cut as short as possible, and then depilated. We set wash‐out period about 2 h until the skin recovered without irradiation or redness. After pentobarbital 40 mg/kg intraperitoneal anesthesia, and alcohol disinfection of the back skin, we irradiated the target area with a power density control at 1.4 ± 5% W/cm^2^ and a spot diameter was 3 mm. The low‐energy group was radiated for 500 mJ while high‐energy group was radiated for 2000 mJ. Thereafter, the guinea pigs were decapitated 24 h postradiation, and then 4 mm (diameter) full‐thickness skin punch biopsies were collected, eluted in RNase‐free water, and immediately put into RNALater Solution for RNA‐Seq assays.

Three guinea pigs were used for pathological assessment. The left back skin was radiated with low energy while the right skin was radiated with high energy. Full‐thickness skin biopsy samples for each parameter were obtained immediately after the THz radiation. Hematoxylin and eosin solution was used to stain the sections. Histological examination and photographs were performed by a light microscope (Olympus IX71).

### 2.3 RNA quantification

RNA integrity was evaluated by the RNA Nano 6000 Assay Kit from the Bioanalyzer 2100 system (Agilent Technologies), RNA integrity number provided by manufacturer protocol suggestion should be more than 9, following the manufacturer’s protocols.

### 2.4 Library preparation

RNA sample preparation for each sample was done using 1 μg of RNA. Briefly, poly‐T oligo‐attached magnetic beads were used to purify messenger RNA. Fragmentation was performed in First Strand Synthesis Reaction Buffer (5X, provided by Beijing Novogene Technology Co., Ltd). First strand cDNA synthesis was done using a random hexamer primer and M‐MuLV Reverse Transcriptase (RNase H−) while DNA Polymerase I and RNase H were used for second strand cDNA synthesis (provided by Beijing Novogene Technology Co., Ltd). Remaining overhangs were transformed into blunt ends through polymerase/exonuclease activities. After the adenylation of 3′ ends of DNA fragments, adaptors with hairpin loop structure were ligated for hybridization. To select the cDNA fragments of preferentially 370–420 bp in length, purification of library fragments was done using the AMPure XP system (Beckman Coulter). Then, polymerase chain reaction (PCR) analyses were performed using Phusion High‐Fidelity DNA polymerase, Index (X, provided by Beijing Novogene Technology Co., Ltd) Primer and Universal PCR primers. PCR products were then purified (AMPure XP system) after which library qualities were assessed on the Agilent Bioanalyzer 2100 system. All the experiments were conducted according to the manufacturers’ instructions.

### 2.5 Quality control

Raw data (raw reads) in the fastq format were first managed via in‐house perl scripts. Here, clean data (clean reads) were acquired by eliminating reads containing adapters, reads1 with poly‐N as well as low‐quality reads from raw data. Moreover, GC, Q20, and Q30 contents from clean data were determined. Downstream analyses were done using high‐quality clean data.

### 2.6 Mapping of the reads to reference genomes

Reference genomes as well as gene model annotation files were directly obtained from the genome database. Reference genome indices were built using Hisat2 v2.0.5. Moreover, aligning of paired‐end clean reads to reference genome was done using Hisat2 v2.0.5. We chose Hisat2 as the mapping tool because it can generate a splice junction database based on gene model annotation file, yielding a better mapping result compared to the other nonsplice mapping tools.

### 2.7 Quantification of gene expression

Read numbers mapped to each gene were counted using feature counts (v1.5.0‐p3; Rsubread [http://www.bioconductor.org]). Then, the Fragments Per Kilobase Million (FPKM) of each gene was calculated based on gene lengths and read counts mapped to the gene. FPKM, expected number of FPKM base pairs sequenced, evaluates sequencing depth and gene length effects on read counts at the same time, and is the most common method for estimating the gene expression level.

The DESeq2 R package (1.20.0) was used to evaluate differential expressions (http://www.bioconductor.org). *p* Values were adjusted through the Benjamini and Hochberg’s approaches to control the false discovery rate. Genes with adjusted *p* < .05 as shown by the DESeq2 were defined as differentially expressed.

### 2.8 Gene Ontology and Kyoto Encyclopedia of Genes and Genomes enrichment analyses of the differentially expressed genes

Cluster Profiler R package (http://www.bioconductor.org) was used to perform Gene Ontology (GO) enrichment analysis of the differentially expressed genes (DEGs). Gene length biases were corrected. Kyoto Encyclopedia of Genes and Genomes (KEGG; https://www.kegg.jp/) is a database for establishing high‐level functions and utilities of biological systems, including cells, organism or the ecosystem. The cluster Profiler R package was used to test statistical significance of DEGs in KEGG pathways.

## 3 RESULTS

### 3.1 Cutaneous response

**FIGURE 1:**
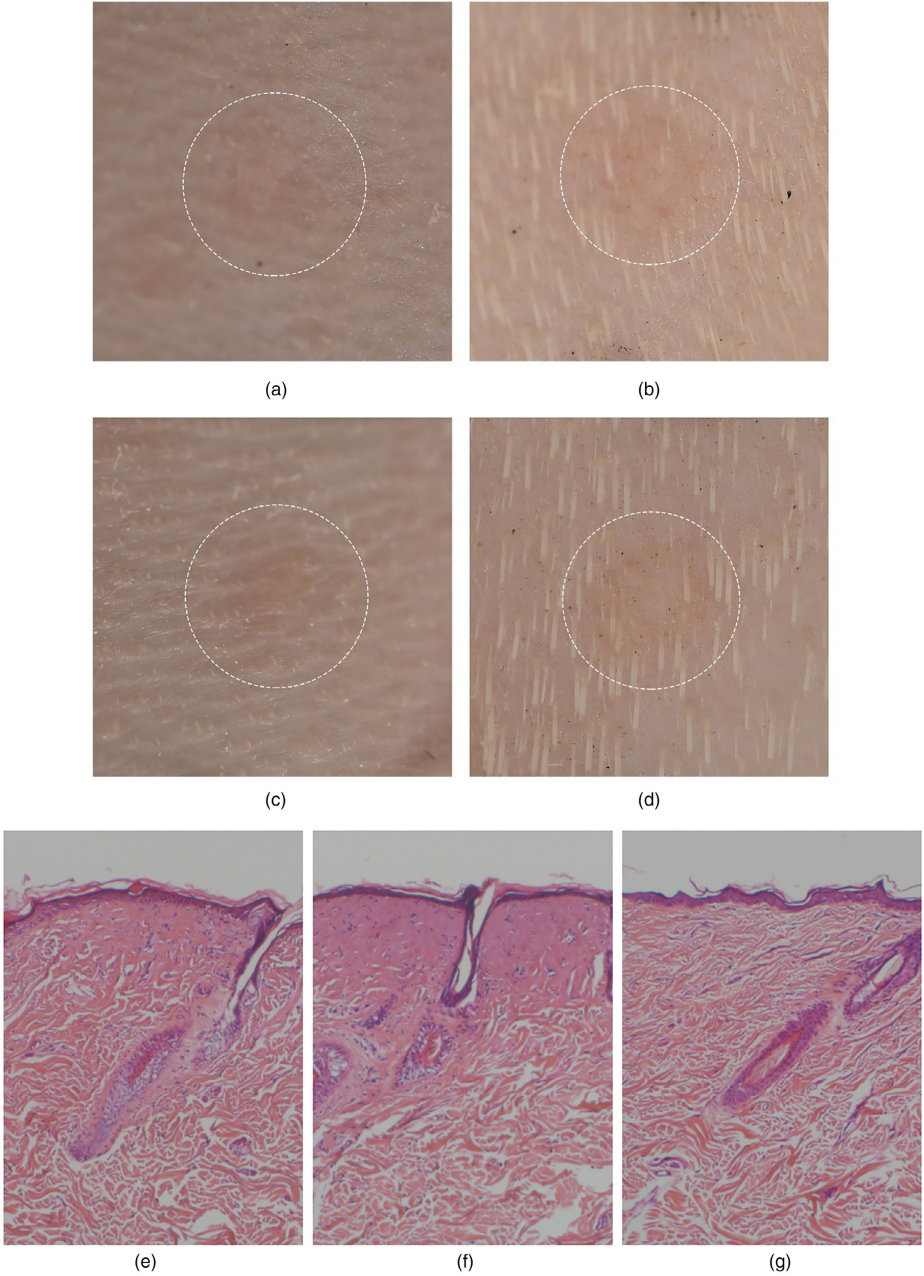
Digital photo after high‐power THz radiation on the guinea pig skin. Broken lines indicate the exposed area (a, low energy, 0 h; b, low energy, 24 h; c, high energy, 0 h; d, high energy, 24 h). Histologic features of guinea pig skin samples (HE stain ×100) at 0 h after high‐power THz radiation with 1.4 W/cm^2^, 3 mm in diameter and a conduction time of 5 or 20 s (e, low energy; f, high energy; g, control). HE, hematoxylin and eosin; THz, terahertz

Subjective histological and imaging evaluation were performed at 0 and 24 h. In the low‐energy group, there was no obvious immediate skin response, while mild erythema was visible at 24 h. In contrast, in high‐energy group, slight erythema and edema were seen at 0 h, while edema disappeared at 24 h and the erythema was more pronounced (Figure 1).

### 3.2 Between the sample correlations

**FIGURE 2:**
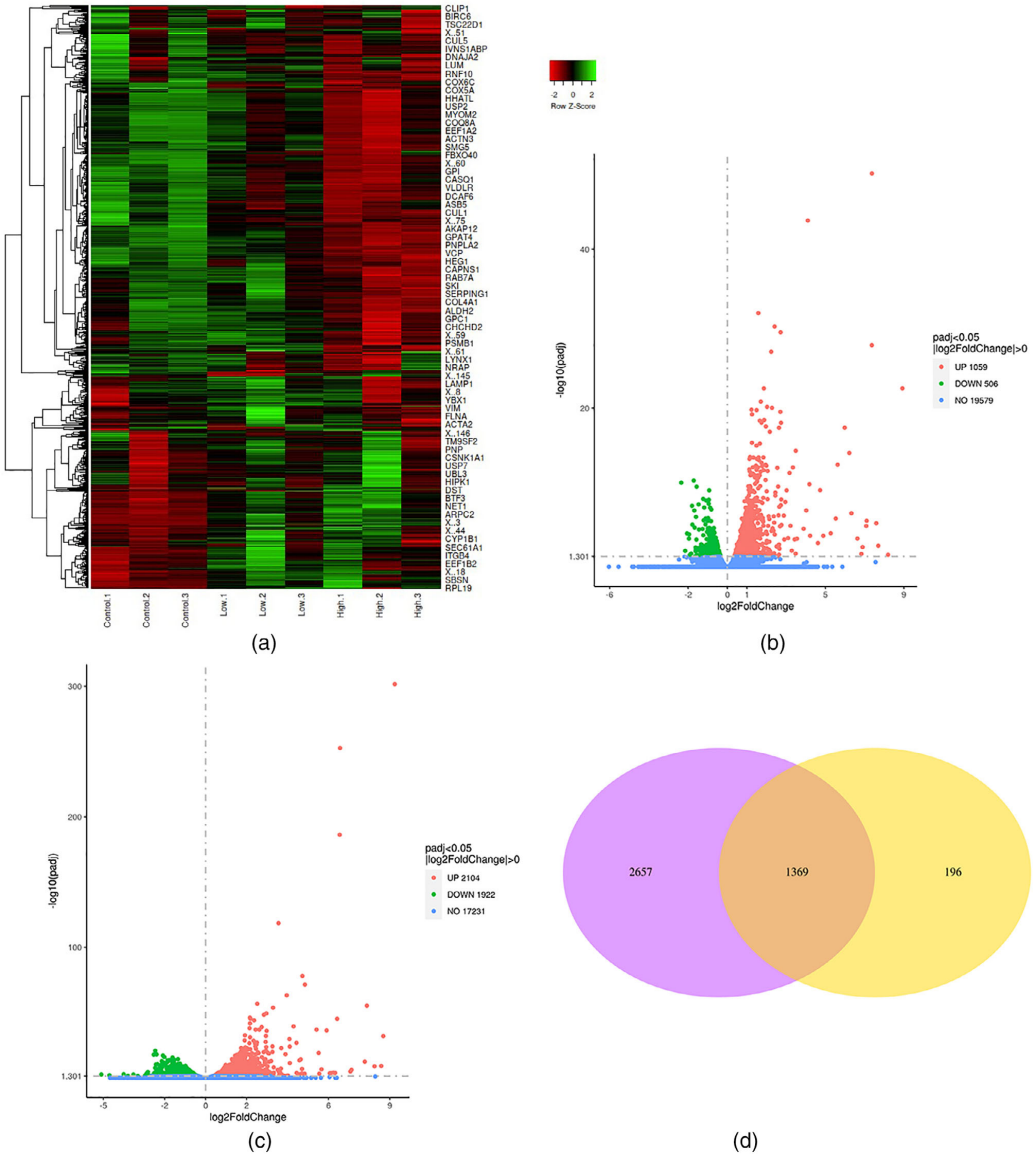
Clustering heat map of the DEGs (a). Abscissa represents sample name, while normalized value of the FPKM differential gene is the ordinate (increasing intensity of the red color is correlated with expression, while an increasing intensity of the green color is correlated with low expression). Volcano plots showing the differential expression of genes. Volcano plots of significantly DEGs, (FDR ≤ 0.05 and |FC| ≥ 1.5; red, upregulated; green, downregulated). Numbers of genes upregulated or downregulated are denoted. Comparison of low energy and control groups (b), comparison of high energy and control groups (c). Common DEGs between low and high energy groups (d). DEG, differentially expressed gene; FPKM, Fragments Per Kilobase Million

Samples from each group were evaluated through hierarchical clustering analysis, and the data showed that three samples in each group exhibited the highest similarity in gene expression (Figure 2). The difference in gene expression was higher between the high‐energy group and the control group compared to that between low‐energy and control groups.

In addition, analysis of the DEGs (DESeq2 *p*_adj_ < .05|log2FoldChange| > 0.0) showed that, 1565 genes were markedly differentially expressed between low‐energy and untreated groups at 24 h postradiation (Figure 2). Out of the 1565, there was upregulation in 1059 genes while 506 were downregulated. On the other hand, 4026 genes were found to be differentially expressed between high‐energy and untreated control groups at 24 h postradiation. Out of the 4026, a total of 2140 genes were found to be upregulated while 1922 were found to be downregulated (Figure 2). Compared to the high‐energy group, the number of DEGs in the low‐energy group was less. Besides, the data showed that there were 1369 common DEGs between low and high‐energy groups (Figure 2).

### 3.3 GO functional enrichment of DEGs

Genes with changed expression responses were shown to mediate a wide range of metabolic and regulatory processes. The clusterProfiler software was used to allocate DEGs in each sample reads into several categories according to their GO terms. GO analysis revealed the number of function related DEGs.

GO functional classification was performed among low‐energy, high‐energy and control group. The top five significantly enriched processes in both the upregulated and downregulated DEGs in the THz‐radiated zone at low‐energy group were skin development, multicellular organismal water homeostasis, establishment of skin barrier, water homeostasis, and regulation of skin‐associated water loss via skin (Table 1). At high energy group, the processes included monocarboxylic acid metabolic process, blood vessel morphogenesis, establishment of skin barrier, blood vessel development, angiogenesis (Table 1).

**TABLE 1:** DEGs and processes enriched in the THz‐radiated zone in the low and high energy group

Group	Description	Number replicates	Fold cut‐off	Differential genes	Upregulated genes	Downregulated genes	BgRatio
Low energy group	Skin development	3	2	47	44	3	165/11,413
	Multicellular organismal water homeostasis	3	2	18	16	2	30/11,413
	Establishment of skin barrier	3	2	13	13	0	16/11,413
	Water homeostasis	3	2	18	16	2	31/11,413
	Regulation of water loss via skin	3	2	13	13	0	17/11,413
	Keratinocyte differentiation	3	2	25	24	1	65/11,413
	Epidermis development	3	2	46	41	5	181/11,413
	Lipid catabolic process	3	2	41	24	17	162/11,413
	Epidermal cell differentiation	3	2	31	28	3	104/11,413
	Lipid biosynthetic process	3	2	60	46	14	310/11,413
High energy group	Monocarboxylic acid metabolic process	3	2	116	45	71	284/11,414
	Blood vessel morphogenesis	3	2	132	49	83	344/11,414
	Establishment of skin barrier	3	2	15	15	0	16/11,414
	Blood vessel development	3	2	153	55	98	416/11,414
	Angiogenesis	3	2	108	42	66	271/11,414
	Skin development	3	2	73	57	16	165/11,414
	Multicellular organismal water homeostasis	3	2	22	18	4	30/11,414
	Vasculature development	3	2	157	57	100	437/11,414
	Regulation of water loss via skin	3	2	15	15	0	17/11,414
	Water homeostasis	3	2	22	18	4	31/11,414

Abbreviations: DEG, differentially expressed genes; THz, terahertz.

### 3.4 KEGG pathway enrichment analysis of the DEGs

**FIGURE 3:**
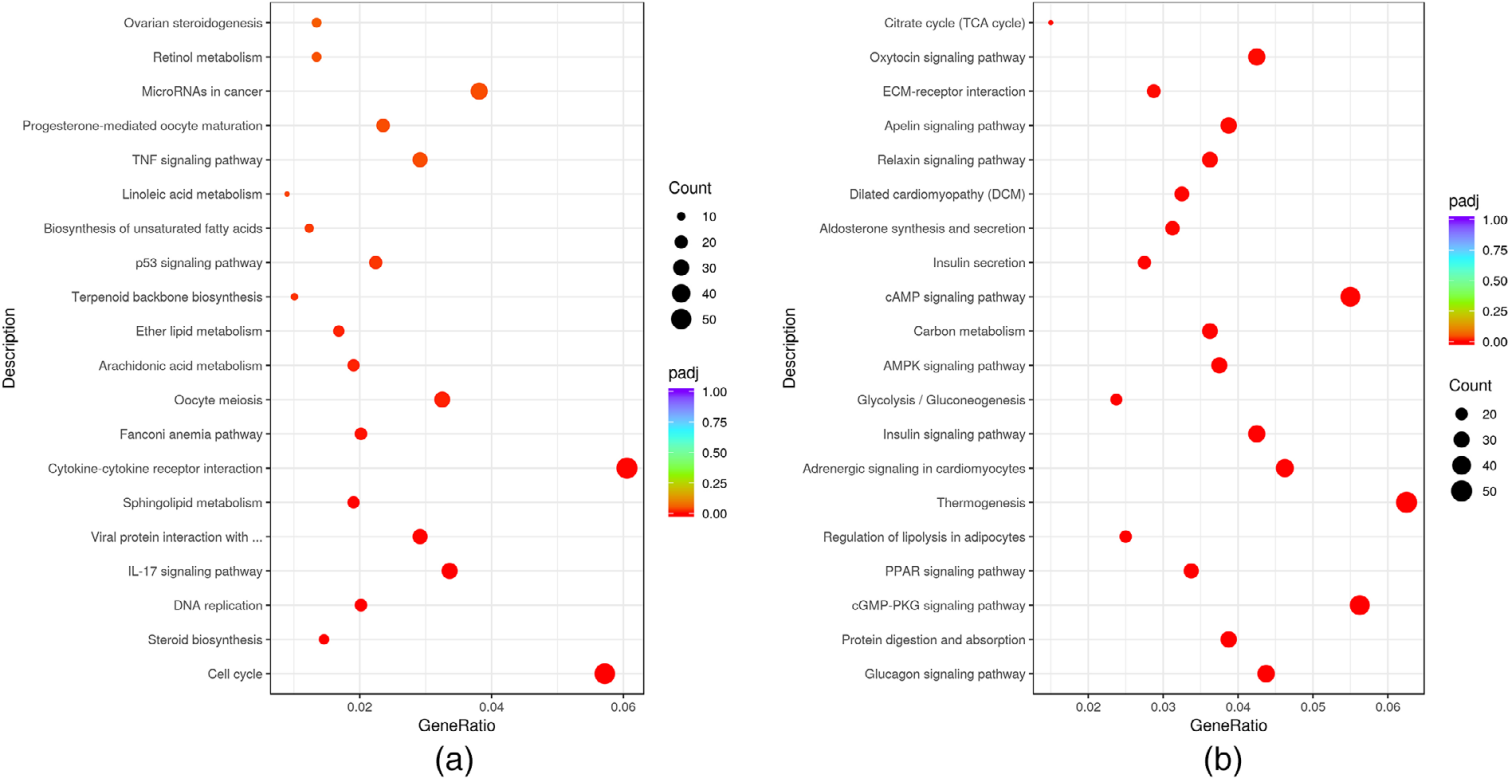
Pathways significantly and differentially enriched in the THz‐radiated zone in the high energy group. The significantly enriched pathways among the upregulated DEGs (a) or the downregulated DEGs (b) in the high energy group. DEG, differentially expressed gene; THz, terahertz

Figure 3 shows the top 20 pathways that were significantly enriched in the upregulated and downregulated DEGs in the high energy group. Significantly enriched pathways among the upregulated DEGs were involved in the cell cycle, steroid biosynthesis, DNA replication, IL‐17 signaling pathway as well as viral protein interactions with cytokine and cytokine receptors. Moreover, the five significantly enriched pathways among the downregulated DEGs were shown to be involved in protein digestion and absorption, glucagon signaling pathway, cGMP‐PKG signaling pathway, PPAR signaling pathway and regulation of lipolysis in adipocytes (Figure 3).

## 4 DISCUSSION

Since the single photon energy of THz radiation is weak because of its long wavelengths, it has good tolerability and a favorable safety profile theoretically. Due to the recent technological development of the THz technique, it has been widely used in many fields including diagnostics of skin melanoma, dysplasia, scars, or security, quality control, artwork examination (Cheng et al., 2021; Ohrstrom et al., 2015). Understanding the interaction between the lasers and tissues enables selection of the most appropriate laser, energy, power, fluence, and irradiance (Carroll and Humphreys, 2006). However, data on the interaction between living skin and the THz sources remains scant and inconsistent (Vilagosh et al., 2019). Since there is more similarity between the skin of guinea pig and humans, it presents a more appropriate small animal model for the short‐term screening of cutaneous radiation injury (Rodgers et al., 2016).

Our preliminary data showed that following a single dose of high‐power THz radiation on living skin, there were trauma, posttraumatic inflammatory reaction, and posttraumatic repair processes. The low‐energy and high‐energy groups showed different cutaneous responses, which were consistent with the different wound depth and inflammatory reaction in the pathological sections. Importantly, the energy we used in our experiment was much higher than the previous THz source, even in the low energy group (Nikitkina et al., 2021).

The thermal effect on the back skin of the guinea pig with a 2.52 THz high‐power optically pumped gas THz source was shown, for the first time, to affect many genes and induce dermal thermal injury in the dermis. Since water strongly absorbs the THz source, it has a great influence on the skin moisture as well as the metabolism and structure of the skin. The thermal effect was more obvious and proportional with the increase in the energy. Thus, the cellular matrix and blood vessels are reconstituted by the thermal effect.

We employed RNA‐seq to screen for the THz‐responsive genes (Wang et al., 2009). The data showed that the low energy THz source mainly affects skin development, multicellular organismal water homeostasis, skin barrier establishment, water homeostasis and skin‐associated water loss regulation. In addition, the top five GO enrichment terms in the higher energy THz source were monocarboxylic acid metabolic process, blood vessel morphogenesis, establishment of skin barrier, blood vessel development, and angiogenesis.

Our data suggest that under high‐energy THz radiation, a few seconds of radiation might affect the skin, compared to the traditional exposure which usually needs up to tens of minutes or several hours (Alexandrov et al., 2013; Franchini et al., 2018). Therefore, the genotoxic properties and THz‐type phototoxicity of the THz range should be reevaluated. Furthermore, there was high expression of genes responsible for water homeostasis, suggesting that intercellular water molecules vibrated when encountering THz irradiation. Besides, the data provides a reference for future studies on the effects of THz sources on the skin, especially in relation to the maintenance of water balance, repair of skin wounds and collagen reconstruction, skin quality improvement, melanin, or lipid metabolism. With the rapid evolution of the THz technology, the high‐power THz source would be widely used in dermatology and cosmetology.

## CONFLICT OF INTERESTS

The authors declare no conflict of interests.

## AUTHOR CONTRIBUTIONS

All authors contributed to the study conception and design. Chengxia Zhou performed the experiment and wrote the manuscript. Lidan Xiong contributed significantly to analysis and manuscript preparation. Xun Zhou helped perform the analysis with constructive discussions. Li Li contributed to the conception of the study. Qiang Yan operate the equipment and wrote the manuscript. All authors read and approved the final manuscript.

## Data Availability

All data included in this study are available upon request by contact with the corresponding author.
